# Potentially Zoonotic *Bartonella* in Bats from France and Spain

**DOI:** 10.3201/eid2303.160934

**Published:** 2017-03

**Authors:** Matthew J. Stuckey, Henri-Jean Boulouis, Florence Cliquet, Evelyne Picard-Meyer, Alexandre Servat, Nidia Aréchiga-Ceballos, Juan E. Echevarría, Bruno B. Chomel

**Affiliations:** University of California, Davis, USA (M.J. Stuckey, B.B. Chomel);; Ecole Nationale Vétérinaire d'Alfort, Maisons-Alfort, France (H.-J. Boulouis);; Agence Nationale de Sécurité Sanitaire de l’Alimentation,de l’Environnement et du Travail (ANSES);; Laboratoire de la Rage et de la Faune Sauvage de Nancy, Malzéville, France (F. Cliquet, E. Picard-Meyer, A. Servat);; Laboratorio de Rabia, Instituto de Diagnóstico y Referencia Epidemiológicos, Mexico City, Mexico (N. Aréchiga-Ceballos);; Centro de Investigación Biomédica en Red de Epidemiología y Salud Pública (CIBERESP);; Instituto de Salud Carlos III, Madrid, Spain (J.E. Echevarría)

**Keywords:** bartonellae, bats, zoonoses, bacteria, France, Spain, *Bartonella* spp

## Abstract

We detected *Bartonella* in 11 of 109 insectivorous bats from France and 1 of 26 bats from Spain. These genetic variants are closely related to bat-associated *Bartonella* described in Finland and the United Kingdom and to *B. mayotimonensis*, the agent of a human endocarditis case in the United States.

Bartonellae have been identified in bats sampled in locations around the world where diverse chiropteran host species can interact with numerous *Bartonella* variants and potential arthropod vectors (*1*–*3*). Many *Bartonella* species are zoonotic, potentially affecting human and bat health (*4*). *Bartonella* spp. in bat populations of Europe are of particular interest because some variants described in Finland and the United Kingdom are closely related to *Bartonella mayotimonensis*, a species detected in the resected aortic valve of a 59-year-old endocarditis patient in the United States (*5*,*6*). To determine if potentially zoonotic bat-associated bartonellae are circulating elsewhere in Europe, we tested insectivorous bats from France and Spain for the presence of *Bartonella* spp. 

We performed necropsies on 26 bats from Spain and 109 from France to collect heart tissue for *Bartonella* spp. diagnostics (Technical Appendix Table 1). Bats from Spain were originally collected during active surveillance for rabies at the Unidad de Aislamiento y Detección Virus, Instituto de Salud Carlos III, Madrid, Spain. Of the bats from France, 97 were originally submitted for passive rabies surveillance to the Agence Nationale de Sécurité Sanitaire de l’Alimentation,de l’Environnement et du Travail (ANSES), Laboratoire de la Rage et de la Faune Sauvage de Nancy in Malzéville, France. We sampled the remaining 12 animals from a rehabilitation center at the Musée d’Histoire Naturelle in Bourges, France. All bats collected for *Bartonella* diagnostics tested negative for rabies. Spatial coordinates were recorded for all bats at the point of sampling before submission for centralized laboratory testing (online Technical Appendix Figure). Whenever possible, we identified bat species and sex. Methods were approved by the University of California, Davis (Davis, CA, USA), Institutional Animal Care and Use Committee (protocol 17669).

We used sampling records to determine the genus and species for 118 of the 135 bats; identification data were not available for 17 of 26 bats from Spain. The 118 identified bats belonged to 8 genera and at least 13 different species: 70 *Pipistrellus* spp. (31 *P. pipistrellus*, 24 *P. nathusii*, 6 *P. kuhlii*, 4 *P. pigmaeus*, and 5 *Pipistrellus* spp.); 15 *Nyctalus noctula*; 7 *Eptesicus serotinus*; 11 *Myotis* spp. (4 *M. mystacinus*, 3 *M. daubentonii*, 1 *M. bechsteinii*, 1 *M. myotis*, 1 *M. nattereri*, 1 *M emarginatus*); 6 *Plecotus* spp. (4 *P. austriacus* and 2 *P. auritus*); 6 *Tadarida teniotis*; 2 *Barbastella barbastellus*; and 1 *Vespertilio murinus*.

We used NucleoSpin Blood QuickPure kits (Machery-Nagel, Düren, Germany) to extract DNA from 25 mg of macerated heart tissue according to the manufacturer’s instructions. We used tissue spiked with *B. henselae* as a positive control for DNA extraction. We screened samples by PCR targeting the citrate synthase gene (*gltA*). Primers CSH1f (GCGAATGAAGCGTGCCTAAA) and BhCS1137.n (AATGCAAAAAGAACAGTAAACA) amplified an ≈350-bp fragment suitable for distinguishing *Bartonella* species (*7*). PCR thermal cycler parameters were set at 10 min at 95°C, followed by 40 cycles of 30 s at 94°C, 1 min at 57°C, 2 min at 72°C, 5 mins at 75°C, and infinite hold at 4°C. We verified amplicon sizes by gel electrophoresis, and Service de Séquençage de Eurofins (Paris, France) generated sequence data from PCR products. 

We used OpenEpi version 3.01 (http://www.openepi.com/) to calculate descriptive statistics and CIs for prevalence data. We constructed phylogenetic trees by using the MrBayes plugin in Geneious version 8.1.7 with a Markov Chain Monte Carlo value of 1,100,000 and 100,000 burn-in length (*8*). We used the ggplot2 package in R (https://www.r-project.org/) to create spatial maps.

We detected *Bartonella* DNA in 12 (8.9%) of 135 bat heart tissue samples (Technical Appendix Table 2); 11 of the tissues were from bats from France, and 1 was from an unidentified bat captured in Torreferrusa, Catalonia, Spain. The 11 *Bartonella*-positive bats from France belonged to only 4 of the 13 sampled species: *N. noctula* (2/15 bats [13.3%, 95% CI 1.7%–40.5%]), P.* nathusii* (6/24 bats [25%, 95% CI 9.8%–46.7%]), *M. daubentonii* (2/3 bats [66.6%, 95% CI 9.4%–99.1%]), and *M. mystacinus* (1/4 bats [25%, 95% CI 0.6%–80.6%]).

All 12 *Bartonella* variants (GenBank accession nos. KY041981–KY041992) clustered closely with zoonotic *B. mayotimonensis* (Figure). Two sequences obtained from *M. daubentonii* bats sampled in Lorraine (GenBank accession no. KY041985) and Upper Normandy (GenBank accession no. KY041989), France, shared 100% nt identity with *Bartonella* strains previously isolated from bats of the same species in Finland and the United Kingdom (*5*,*9*). None of the *Bartonella* variants were closely related to *Candidatus* Bartonella naantaliensis or *Candidatus* Bartonella hemsundetiensis, which were also described in bats sampled in Finland (*5*,*10*). The absence of variants resembling these bartonellae from northern Europe suggests a spatial heterogeneity in the distribution of *Bartonella* spp. across bat populations and selective adaptations to specific host reservoirs. 

**Figure:**
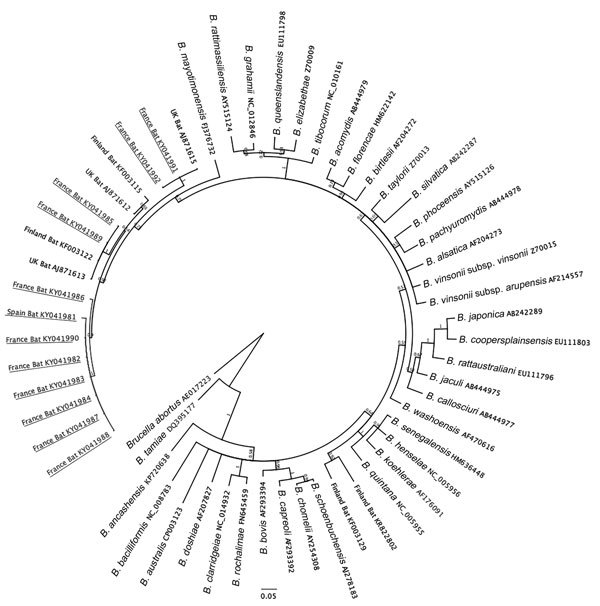
Phylogenetic analysis of citrate synthase (*gltA*) gene sequences of 12 *Bartonella* spp. variants detected in bats from France and Spain (underlined) compared with sequences from GenBank. All 12 of these variants clustered with zoonotic *B. mayotimonensis.*

Further research is needed to better evaluate the prevalence of zoonotic *Bartonella* species in western Europe and to determine if *B. mayotimonensis*, the agent of a US case of human endocarditis, is present across a broader range than currently documented. Future studies should consider specifically focusing on *Nyctalus, Pipistrellus,* and *Myotis* bat species, from which we most frequently detected variants similar to *B. mayotimonensis*.

Technical Appendix Geographic origins of bats samples in France and Spain and descriptive characteristics of *Bartonella*-positive bats.
