# Supplementary material for: Potentially Zoonotic Bartonella in Bats from France and Spain
Source: Emerg Infect Dis. 2017 Mar;23(3):539–41. doi: 10.3201/eid2303.160934 (PMC5382759; doi:10.3201/eid2303.160934)
Supplement: Technical Appendix — Geographic origins of bats samples in France and Spain and descriptive characteristics of Bartonella-positive bats. [file 16-0934-Techapp-s1.pdf]

Article DOI: http://dx.doi.org/10.3201/eid2303.160934

Potentially Zoonotic *Bartonella* in Bats from France and Spain

Technical Appendix

Technical Appendix Table 1. Number of bats sampled in France and Spain, by geographic origin

Country and Region	Number
France	109
Alsace	34
Lorraine	23
Normandie	14
Brittany	12
Rhône-Alpes	8
Aquitaine	5
Centre	4
Champagne-Ardenne	4
Franche-Comté	1
Ile de France	1
Midi-Pyrénées	1
Pays de Loire	1
Poitou-Charentes	1
Spain	26
Catalonia	22
Andalusia	3
Madrid	1

Technical Appendix Table 2. Descriptive characteristics of the 12 *Bartonella* positive bats from France and Spain*

Study ID	Species	Sex	Location	Region	Country	GenBank accession no.
NC21	*Myotis daubentonii*	M	Duclair	Upper Normandy	France	KY041989
NC82	*Myotis daubentonii*	F	Sorcy-Saint-Martin	Lorraine	France	KY041985
NC45	*Myotis mystacinus*	M	Novéant-sur-Moselle	Lorraine	France	KY041986
BR05	*Nyctalus noctula*	M	Andernay	Lorraine	France	KY041992
BR09	*Nyctalus noctula*	M	Andernay	Lorraine	France	KY041991
NC09	*Pipistrellus nathusii*	F	Épizon	Champagne-Ardenne	France	KY041990
NC35	*Pipistrellus nathusii*	F	Colmar	Alsace	France	KY041988
NC44	*Pipistrellus nathusii*	M	Metz	Lorraine	France	KY041987
NC89	*Pipistrellus nathusii*	M	Saint-Martin-le-Gaillard	Upper Normandy	France	KY041984
NC92	*Pipistrellus nathusii*	M	Aingoulaincourt	Champagne-Ardenne	France	KY041983
NC93	*Pipistrellus nathusii*	F	Bertignolles	Champagne-Ardenne	France	KY041982
SP08	ND	ND	Torreferrusa	Catalonia	Spain	KY041981

*ND, not determined.

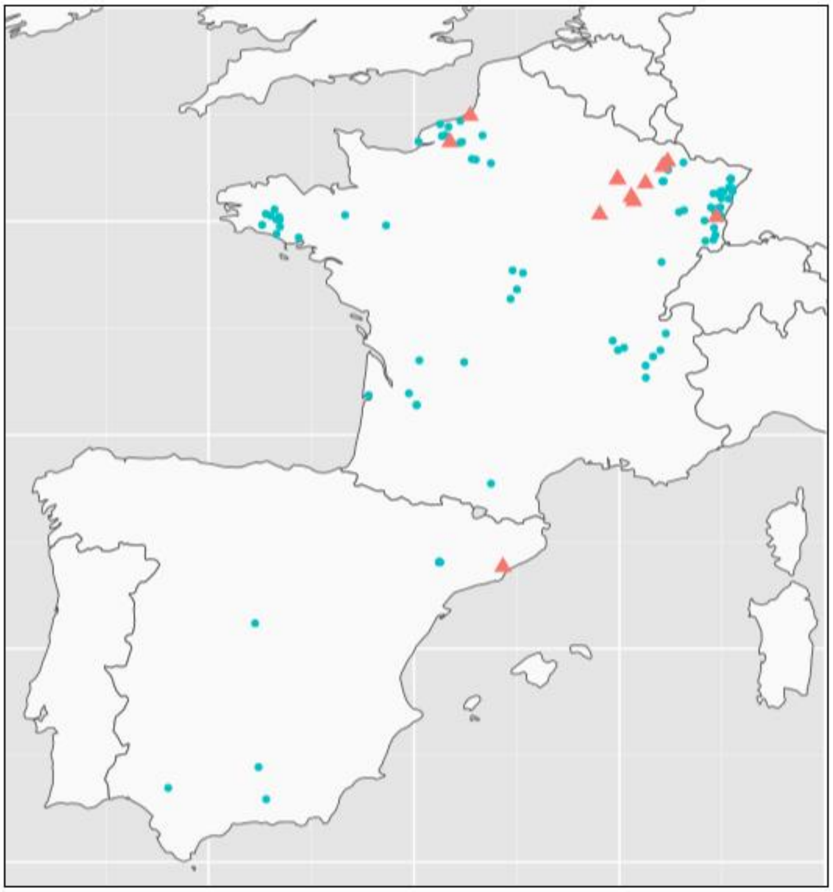

Technical Appendix Figure. Coordinates of bats sampled in the France and Spain. Red triangles illustrate locations of animals that tested positive for *Bartonella*, blue circles indicate negative samples.
